# Timing of Primary Tooth Eruption in Infants Observed by Their Parents

**DOI:** 10.3390/children10111730

**Published:** 2023-10-25

**Authors:** Mina Dodo, Chiharu Ota, Motohiro Ishikawa, Ichie Koseki, Junichi Sugawara, Nozomi Tatsuta, Takahiro Arima, Nobuo Yaegashi, Takeyoshi Koseki

**Affiliations:** 1 Division of Preventive Dentistry, Tohoku University Graduate School of Dentistry, Sendai 980-8575, Japan; mina.dodo.c1@tohoku.ac.jp (M.D.); yobou@dent.tohoku.ac.jp (M.I.); ichie.koseki.c1@tohoku.ac.jp (I.K.); takeyoshi.koseki.b6@tohoku.ac.jp (T.K.); 2 Development and Environmental Medicine, Tohoku University Graduate School of Medicine, Sendai 980-8575, Japan; nozomi.tatsuta.a5@tohoku.ac.jp; 3 Department of Pediatrics, Tohoku University Hospital, Sendai 980-8574, Japan; 4 Environment and Genome Research Center, Tohoku University Graduate School of Medicine, Sendai 980-8575, Japan; junichi.sugawara.c3@tohoku.ac.jp (J.S.); takahiro.arima.a1@tohoku.ac.jp (T.A.); nobuo.yaegashi.c7@tohoku.ac.jp (N.Y.); 5 Department of Obstetrics and Gynecology, Tohoku University Graduate School of Medicine, Sendai 980-8575, Japan

**Keywords:** tooth eruption, child development, cohort studies

## Abstract

Background: The timing of primary teeth eruption is a visible indicator of infant physical growth other than body weight or height. It also reflects neurological integrity and development as well as nutrition, socioeconomic state, or underlying diseases. Therefore, the timing of primary teeth eruption is one of the major concerns for parents in health checkups for infants and children. However, the detailed developmental timing of teeth eruption differs depending on the survey methodology, country, or generation. We hypothesized that the timing of primary teeth eruption differs between the medical checkup by dentists and the daily records by parents. Methods: We conducted a questionnaire survey on the date of eruption of primary teeth as an adjunct study among Miyagi Regional Center participants in the Japan Environment and Children’s Study (JECS), a large-scale birth cohort study. A total of 1695 responses (3793 participants) were analyzed. Results: The median ages of eruption were 7.1 months (male) and 7.6 months (female) for mandibular primary central incisors, 8.7 months (male) and 9.2 months (female) for maxillary primary central incisors, 10.0 months (male) and 10.3 months (female) for maxillary primary lateral incisors, and 10.4 months (male) and 10.8 months (female) for mandibular primary lateral incisors, which were earlier than the reported timings based on dental check-ups. Comparing the eruption time of preterm and term infants, the eruption time was earlier in preterm infants in the corrected ages. Conclusions: The eruption timing observed and described by the parents is earlier than that examined by dentists at regular check-ups. In addition to examining the primary teeth eruption of full-term birth children, we also examined that of preterm birth children because of the increasing number of premature births. To the best of our knowledge, this is the first report from a large cohort study to clarify the eruption time of primary teeth monitored by parents.

## 1. Introduction

Body weight and height are commonly used to evaluate the developmental and nutritional status of infants. In addition, the eruption time of primary teeth, i.e., deciduous teeth, such as primary central incisors and primary lateral incisors, is a visible indicator of infant physical growth other than body weight or height. It also reflects neurological integrity and development as well as nutrition, socioeconomic state, or underlying diseases. Therefore, the timing of primary teeth eruption is one of the major concerns for parents in health checkups for infants and children [1]. The Japanese Society of Pediatric Dentistry (JSPD) conducted nationwide surveys, based on regular check-ups dentists (every 1 months), on the eruption time of primary teeth in 1984 [2] and 2019 [3]. For primary teeth, the average age of eruption of mandibular primary central incisors changed from 8.0 months in 1984 to 6.8 months in 2019 for males and from 9.0 to 7.5 months for females, and that of maxillary primary central incisors changed from 10.0 to 8.9 months for males and from 10.0 to 9.4 months for females, indicating an acceleration in the age of eruption over 35 years [2,3]. For mandibular primary lateral incisors, the age changed from 12.0 to 11.8 in males and 12.0 to 11.9 in females, and for maxillary primary lateral incisors, it changed from 11.0 to 11.1 in males and 11.0 to 11.0 in females [2,3]. Thus, it was speculated that the date of primary tooth eruption varied greatly depending on the generation of the children because of nutrition, lifestyle, or environmental changes [4]. The examinations were performed by the participating dentists in previous studies. In general, it is recommended that dental check-ups for infants should be started within 6 months from the first eruption of their teeth [1] or their first birthdays. Therefore, it might be difficult to determine the day-to-day changes in infantile dental development during a check-up by dentists. In contrast, parents observed the status and timing of tooth eruption in their children daily. However, the eruption time of the primary tooth observed and reported by parents has not yet been studied.

The Japan Environment and Children’s Study (JECS), a prospective, ongoing birth cohort study with approximately 100,000 children and their parents throughout Japan, from pregnancy to age 13, has been conducted since 2011 [5,6]. The goal of the JECS is to elucidate how the environment affects children’s health. It covers environmental, genetic, social, and lifestyle factors as well as detailed biochemical tests, and examines health and developmental status. We conducted this adjunct study of the JECS with 9217 mother–infant pairs at the Miyagi Regional Center. An additional questionnaire survey on oral development including the timing of primary tooth eruption was conducted for the 3793 participants who agreed to join this adjunct study.

In the present study, we hypothesized that the eruption time observed and described by the parents was earlier than that examined by dentists at regular check-ups. Therefore, we aimed to examine the eruption times of the primary teeth of infants observed and described in the questionnaire by their parents and to assess their relationships with age. In addition to examining the primary teeth eruption of full-term birth children, we examined whether prematurity or low birth weight affects the eruption time. To the best of our knowledge, this is the first report from a large cohort study to clarify the eruption time of primary teeth monitored by parents.

## 2. Materials and Methods

### 2.1. Study Population

This was an adjunct study of the JECS in the Miyagi Regional Center. The study design of the JECS has been described previously [3]. In brief, pregnant women were recruited from 15 regional centers, including the Miyagi Regional Center, from January 2011 to March 2014. In the Miyagi Regional Center, 9217 participants were registered from 2011 to 2014 in 14 municipalities in the Miyagi Prefecture. The present study, entitled “the cohort study on healthy oral development and disease prevention”, is an adjunct study of the JECS in the Miyagi Regional Center with 3793 out of 9217 participants who agreed to join the study. Written informed consent was obtained from all the participants.

### 2.2. Methods

**Figure 1:**
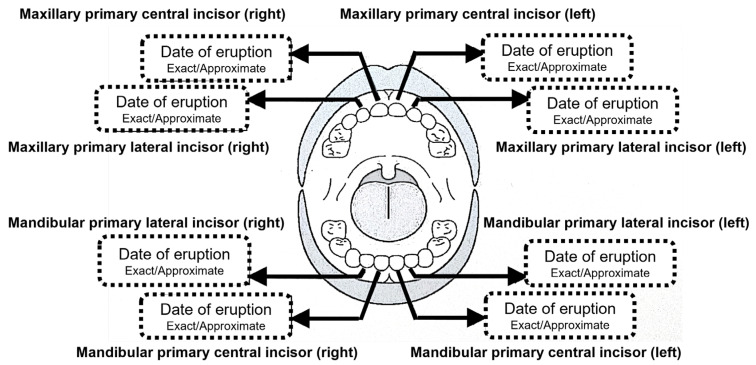
A diagram of the dentition in the questionnaire.

The questionnaire, in Japanese, was administered twice between 2013 and 2016: at 1.5 years of age and at 3.5 years of age. A diagram of the dentition was shown on the questionnaire (Figure 1), and the respondents were asked to indicate the month and date of eruption of the primary teeth, such as the maxillary and/or mandibular primary central incisors and maxillary and/or mandibular primary lateral incisors. The respondents were asked to indicate whether the date of eruption was “exact” or “approximate”. If a non-numerical entry, such as “in the early”, “in the middle”, or “in the late” of the month, was made in the “month-day” entry field, they were converted to the 5th, 15th, or 25th day of the month, respectively. In the same way, entries such as “the beginning of the month” and “the end of the month were converted to the 1st and 30th day, respectively. Answers were excluded if they were inconsistent, that is, when five or more teeth erupted on the same day, when the eruption dates differed between the two surveys, or when the primary lateral incisors erupted earlier than the primary central incisors in the same jaw.

### 2.3. Definitions

Gestational age is defined as time elapsed between the first day of the last menstrual period and the day of delivery. Preterm delivery is defined as babies born alive before 37 weeks of gestational age [7]. Chronological age is defined as the age from birth. Further, in preterm babies, corrected age was calculated as the chronological age in weeks minus the number of weeks preterm at birth [8]. Low birth weight is defined as a weight at birth of less than 2500 g [7].

### 2.4. Statistical Analysis

IBM SPSS Statistics Ver. 21.0 (IBM Japan, Tokyo, Japan) was used for statistical analysis. Mann–Whitney U tests were used to compare differences between preterm and full-term or low- and normal-weight infants. The *p*-value was set at *p* < 0.05.

## 3. Results

**Figure 2:**
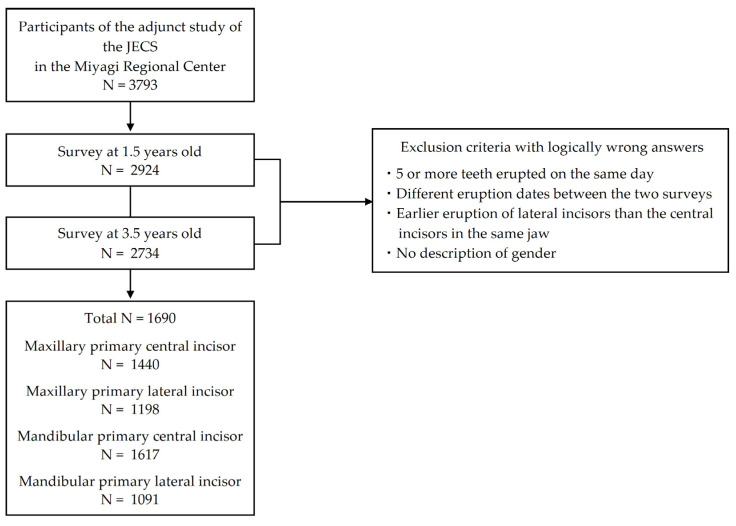
Flow diagram of this adjunct study of the JECS in the Miyagi Regional Center.

Of the 3793 participants who agreed to join the adjunct study in the beginning, 2924 responded for the 1.5-year-old surveys and 2734 participants responded for the 3.5-year-old surveys. The total number of participants was 1690, with 839 males and 851 females. Of these, we analyzed 1440 maxillary primary central incisors, 1198 maxillary primary lateral incisors, 1617 mandibular primary central incisors, and 1091 mandibular primary lateral incisors (Figure 2).

The mean maternal age was 30.6 years, and 41% (692/1690 participants) were first-time mothers. The median gestational period was 37.3 weeks, with 6.2% (152/1686, 4 were excluded because of no description) being preterm births (<37 weeks of gestation). The mean birthweight was 3020 g, with 9.0% (105/1686, 4 were excluded because of no description) having a low birth weight (<2500 g) (Table 1). Using chronological age, the median ages of eruption were 8.7 months (266 days) (male) and 9.20 months (280 days) (female) for maxillary primary central incisors, 10.0 months (304 days) (male) and 10.3 months (315 days) (female) for maxillary primary lateral incisors, 7.1 months (216 days) (male) and 7.6 months (231 days) (female) for mandibular primary central incisors, and 10.4 months (316 days) (male) and 10.8 months (328 days) (female) for mandibular primary lateral incisors (Table 2). Regarding sex differences, males had significantly earlier eruption dates than females for mandibular primary central incisors and maxillary primary lateral incisors. The sex difference in the median eruption date was 15.0 days for mandibular primary central incisors, 14.0 days for maxillary primary central incisors, 9.5 days for maxillary primary lateral incisors, and 11.5 days for mandibular primary lateral incisors.

**Figure 3:**
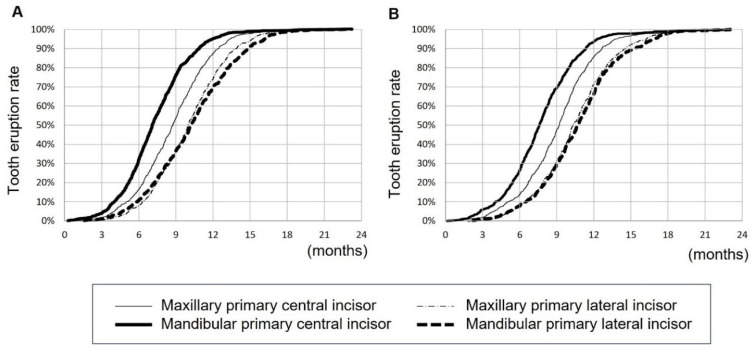
Timing of primary teeth eruption in (**A**) males and (**B**) females from birth (chronological age).

Next, we compared the eruption times between preterm and full-term infants. We found no significant difference in the chronological age between preterm and full-term infants. On the other hand, in terms of gestational age, the eruption time was significantly earlier in preterm than in full-term infants in maxillary primary central incisors, maxillary primary lateral incisors, mandibular primary central incisors, and mandibular primary lateral incisors in males and maxillary primary central incisors and maxillary primary lateral incisors in females (Table 3). In summary, the eruption time was earlier in preterm infant in terms of corrected ages. Figure 3 shows the sigmoid curve graphs of the timing of primary teeth eruption in males and females from birth using chronological age. Further, compared to the previous reports [3,4], we found that the eruption timings were mostly earlier in the present study, except for the timing of mandibular primary central incisors in the study of 2019 [4] (Table 4).

## 4. Discussion

In the present study, we showed that eruption timing of primary teeth observed and described by the parents is earlier than that examined by dentists at regular check-ups. The most common method for estimating the eruption date is probit analysis [9], which was used in the JSPD study [3,4]. This method was used to approximate the normal distribution model by adapting the cumulative tooth eruption rate curve, which was created by collecting each oral examination. The difference between the true eruption date and the observation date when the oral examination was performed by the dentists was logically corrected by making an accurate approximation [10]. In contrast, in this study, the eruption time of primary teeth was recorded in the questionnaire by the parents. Since parents provide childcare every day, they are likely to observe the emergence of the tooth earlier than if they were to confirm the emergence on the date of the oral examination. Various studies have reported the factors related to the primary tooth eruption time [11,12,13]; factors such as gender and race [14,15,16,17,18,19,20,21], socioeconomic status [22], birth weight and postnatal development [17,23], nutritional status [19,24,25,26], and congenital endocrine and metabolic diseases such as hypothyroidism [19,27] have been considered to be associated with the eruption time of the primary tooth. Therefore, the accurate estimation of the primary tooth eruption is important to follow the neurological integrity, physical development, nutrition, socioeconomic state, or underlying diseases of children. The sigmoid curve we made in the present study (Figure 3) will help parents/dentists to estimate the eruption date of children.

In general, preterm infants tend to show delayed development, including eruption time of primary teeth in terms of chronological age [28,29,30]. However, the present study suggests that the eruption time of primary teeth did not differ in terms of chronological age between preterm and full-term infants, but was earlier in preterm infants in terms of gestational age, which means the eruption time was earlier in preterm infant in terms of corrected ages. The growth curve by gestational age in [31] shows that head circumference growth increases similarly to height and weight growth up to 37 weeks, but after 40 weeks, head circumference, height, and weight growth do not increase. Scammon’s organ-specific growth curves [32] show that after birth, the development of the head, which is included in the neural type, precedes the development of other organ types, suggesting that tooth eruption to maintain feeding following suckling milk may also be determined by the period from birth. In another study, it is reported that the average eruption in preterm children with birth weight small for gestational age was earlier than those appropriate for gestational age [12]. Intrauterine nutritional changes might be associated with the eruption timing.

This study had some limitations. Since this study was based on intraoral observations of parents, it is possible that the eruption time of the primary teeth was less accurately identified and recorded. However, in this study, we asked the parents to describe only primary central incisors and primary lateral incisors, which are easy to find and reported to erupt earlier than the primary canines and molars [33]. In addition, since our study is an adjunct study of a national large-cohort study, large numbers of parents participated in our survey. Further, we excluded the possibly wrong answers such as 5 or more teeth erupted on the same day, different eruption dates between the two surveys, or earlier eruption of lateral incisors than the central incisors in the same jaw according to the past report [33] and our own clinical experience. Therefore, we believe that the accuracy of the information can be assured in the present study. Another limitation is that the results of this study would have been more accurate if the survey had been conducted earlier, including when the primary central incisors were erupting.

## 5. Conclusions

We investigated the eruption times of maxillary primary central incisors, maxillary primary lateral incisors, and mandibular primary lateral incisors observed by parents. The sigmoid curve we made in the present study will help parents/dentists/healthcare professionals to estimate the eruption dates of children’s teeth. The accurate estimation of the primary tooth eruption is important to evaluate the neurological integrity, physical development, nutrition, socioeconomic state, or underlying diseases of children.

## Figures and Tables

**Table 1:** Characteristics of the participants.

	Total	Gender	
		Male	Female
	1690	839	851
Maternal age at birth (years old)			
–20	17	6	11
21–25	234	115	119
26–30	590	285	305
31–35	550	283	267
36–40	248	125	123
41–	39	21	18
Unknown	12	4	8
Mean ± SD (years old)	30.6 ± 4.9	30.8 ± 4.9	30.5 ± 4.9
Median (years old)	29	29	29
Gestational weeks (weeks)			
–31	21	13	8
32–36	84	49	35
37–	1581	776	805
Unknown	4	1	3
Mean ± SD (weeks)	37.1 ± 1.9	36.9 ± 2.0	37.2 ± 1.8
Median (weeks)	37.3	37.2	37.2
Preterm birth (<37 weeks)	6.2%	7.4%	5.1%
Birth weight (g)			
–999	11	5	6
1000–1499	7	6	1
1500–2499	134	63	71
2500–3999	1513	752	761
4000–	21	12	9
Unknown	4	1	3
Mean ± SD (g)	3020 ± 508	3058 ± 501	2997 ± 468
Median (g)	3052	3088	3088
Low birth weight (<2500 g)	9.0%	8.8%	9.2%

**Table 2:** Eruption timings of primary teeth in males and females in chronological ages.

Gender	Tooth	N	Mean ± SD	10 Percentiles	Median	90 Percentiles
Male	Chronological age (months)					
	maxillary primary central incisor	709	8.7 ± 1.8	5.1	8.7	12.6
	maxillary primary lateral incisor	588	10.0 ± 1.9	6.4	10.0	14.0
	mandibular primary central incisor	807	7.4 ± 1.8	4.1	7.1	11.1
	mandibular primary lateral incisor	545	10.5 ± 2.2	6.1	10.4	15.3
Female	Chronological age (months)					
	maxillary primary central incisor	731	9.1 ± 2.0	5.0	9.2	12.9
	maxillary primary lateral incisor	610	10.5 ± 2.0	6.5	10.3	14.7
	mandibular primary central incisor	810	7.7 ± 1.9	4.1	7.6	11.5
	mandibular primary lateral incisor	546	10.8 ± 2.1	6.6	10.8	15.6

**Table 3:** Eruption timings of primary teeth in normal and preterm infants in chronological and gestational ages (*: *p* values less than 0.05).

Gender	Tooth	Delivery	Chronological Age (Months)			Gestational Age (Months)		
			N	Mean ± SD	*p*	N	Mean ± SD	*p*
**Male**	maxillary primary central incisor	All	704	8.7 ± 2.9		704	17.7 ± 2.9	
		Full-term	652	8.8 ± 2.9	0.971	652	17.8 ± 2.9	0.006 *
		Preterm	52	8.6 ± 2.7		52	16.5 ± 2.9	
	maxillary primary lateral incisor	All	585	10.0 ± 3.0		585	19.0 ± 3.0	
		Full-term	545	10.0 ± 3.0	0.723	545	19.1 ± 3.0	0.048 *
		Preterm	40	10.1 ± 3.0		40	17.9 ± 3.1	
	mandibular primary central incisor	All	802	7.4 ± 2.9		802	16.3 ± 2.9	
		Full-term	742	7.4 ± 2.9	0.841	742	16.5 ± 2.9	0.001 *
		Preterm	60	7.2 ± 2.6		60	15.0 ± 2.9	
	mandibular primary lateral incisor	All	541	10.5 ± 3.5		541	19.5 ± 3.6	
		Full-term	504	10.6 ± 3.5	0.261	504	19.6 ± 3.5	0.006 *
		Preterm	37	9.6 ± 3.8		37	17.5 ± 3.9	
**Female**	maxillary primary central incisor	All	725	9.1 ± 3.1		725	18.2 ± 3.2	
		Full-term	697	9.2 ± 3.1	0.213	697	18.3 ± 3.2	0.002 *
		Preterm	28	8.2 ± 3.1		28	16.2 ± 3.3	
	maxillary primary lateral incisor	All	608	10.5 ± 3.2		608	19.5 ± 3.3	
		Full-term	585	10.5 ± 3.2	0.448	585	19.6 ± 3.2	0.033 *
		Preterm	23	10.1 ± 3.4		23	18.2 ± 3.5	
	mandibular primary central incisor	All	804	7.7 ± 3.0		804	16.7 ± 3.1	
		Full-term	766	7.7 ± 3.0	0.519	766	16.8 ± 3.0	0.053
		Preterm	38	8.0 ± 3.2		38	15.8 ± 3.3	
	mandibular primary lateral incisor	All	541	10.8 ± 3.4		541	19.8 ± 3.4	
		Full-term	521	10.8 ± 3.4	0.675	521	19.9 ± 3.4	0.079
		Preterm	20	10.3 ± 3.4		20	18.3 ± 3.6	

**Table 4:** Comparison of eruption timings of primary tooth across studies.

Published Year	2022 *	2019 [4]	1988 [3]
Method	Self-Record	Dental Check-Ups	Dental Check-Ups
Total number	1695	1379	845
Chronological age (months)	Mean ± SD	Mean ± SD	Mean ± SD
Male			
maxillary primary central incisor	8.7 ± 1.8	8.9 ± 1.8	10.0 ± 1.0
maxillary primary lateral incisor	10.0 ± 1.9	11.1 ± 2.5	11.0 ± 1.0
mandibular primary central incisor	7.4 ± 1.8	6.8 ± 2.1	8.0 ± 1.0
mandibular primary lateral incisor	10.5 ± 2.2	11.8 ± 3.2	12.0 ± 2.0
Female			
maxillary primary central incisor	9.1 ± 2.0	9.4 ± 1.9	10.0 ± 1.0
maxillary primary lateral incisor	10.5 ± 2.0	11.0 ± 2.0	11.0 ± 2.0
mandibular primary central incisor	7.7 ± 1.9	7.5 ± 1.9	9.0 ± 1.0
mandibular primary lateral incisor	10.8 ± 2.1	11.9 ± 2.5	12.0 ± 2.0

* Present study.

## Data Availability

The datasets generated and/or analyzed during the current study are available from the corresponding author on reasonable request.
